# Metabolic syndrome and venous thromboembolism: the role of abdominal obesity and sex differences

**DOI:** 10.1007/s11239-026-03255-x

**Published:** 2026-03-06

**Authors:** Niklas Brodin, Peter Nymberg, Beata Borgström Bolmsjö, Peter J. Svensson, Johan Elf, Bengt Zöller, Susanna Calling

**Affiliations:** 1 University Clinic Primary Care, Skåne University Hospital, Region Skåne, Malmö, 205 02 Sweden; 2 Center for Primary Health Care Research, Department of Clinical Sciences, Lund University, Jan Waldenströms Gata 35, Malmö, Sweden; 3 Department of Hematology, Oncology and Radiotheraphy, Skåne University Hospital, and Departement of Translational Research, Lund University, Both Malmö, Sweden

**Keywords:** Abdominal obesity, Deep vein thrombosis, Metabolic syndrome, Pulmonary embolism, Venous thromboembolism

## Abstract

Metabolic syndrome (MetS) is a cluster of cardiovascular risk factors, including abdominal obesity, insulin resistance, dyslipidemia, and hypertension. Its association with venous thromboembolism (VTE) has been inconsistently reported. We investigated the association between MetS and VTE, and the influence of sex and abdominal obesity. We included 12,788 middle-aged and older adults from the re-examination phase of the Swedish prospective cohort Malmö Preventive Project (2002–2006). VTE events were collected using national registers until 31 December 2018. MetS was defined as the presence of three or more of the following factors: abdominal obesity, elevated triglycerides, low high-density lipoprotein cholesterol, hypertension, and impaired fasting glucose. During a mean follow-up of 11 years, 730 first VTE events occurred. The prevalence of MetS was higher in men than in women (*p* < 0.001). In analyses adjusted for age, height, and smoking, MetS was not associated with increased risk of VTE in men (HR 1.10; 95% CI 0.90–1.34) or women (HR 1.15; 95% CI 0.91–1.45). In women, higher VTE risk was found in those with multiple MetS factors compared to zero. Abdominal obesity was associated with VTE risk in women only (HR 1.80; 95% CI 1.43–2.27). MetS was not associated with VTE risk in men or women. The higher VTE risk observed with multiple MetS components in women appeared largely attributable to abdominal obesity, with little contribution from other components. Risk factors for VTE may differ by sex, with abdominal obesity appearing particularly relevant for women.

**Graphical Abstract:**

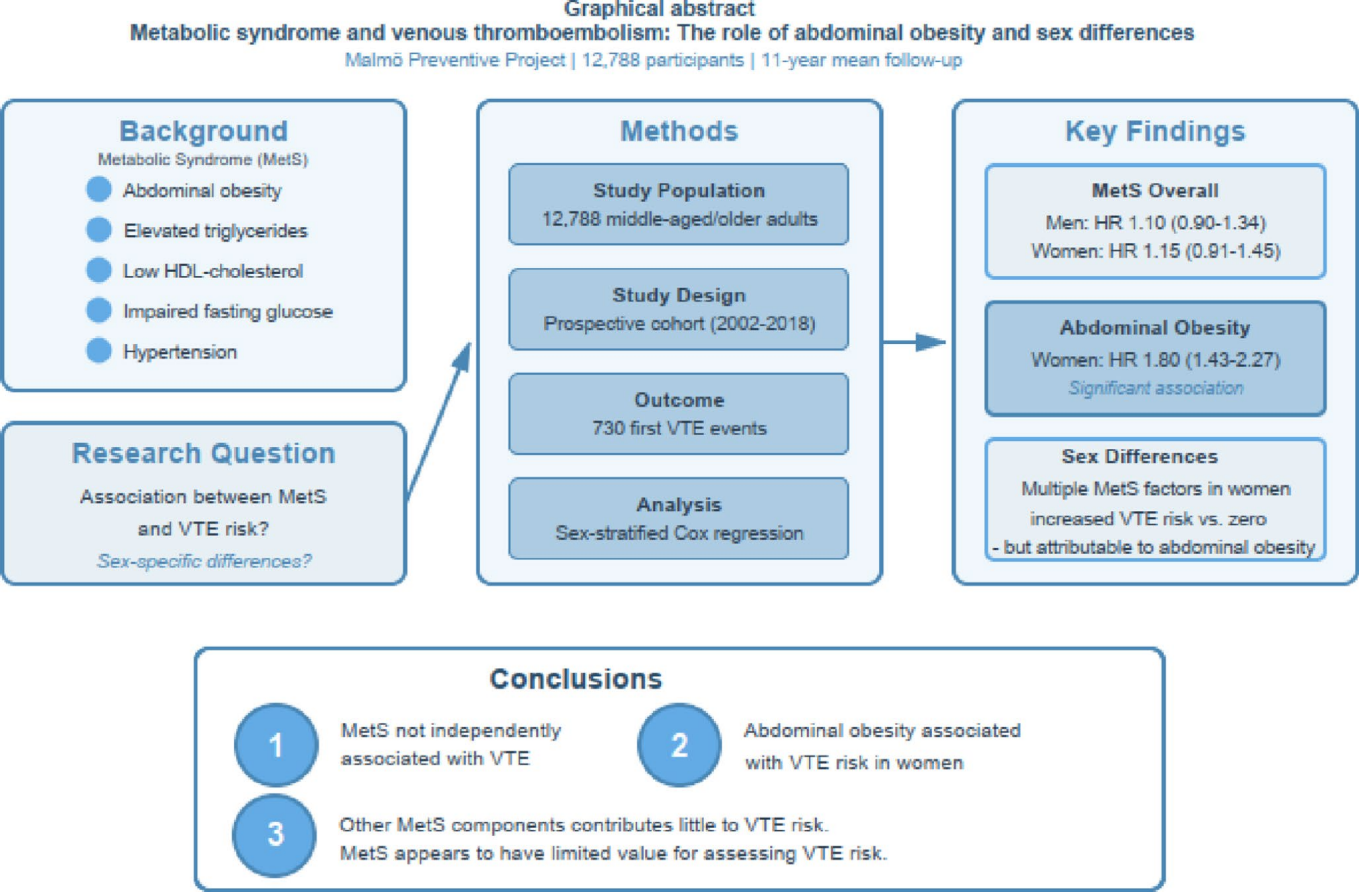

**Supplementary Information:**

The online version contains supplementary material available at 10.1007/s11239-026-03255-x.

## Introduction

Venous thromboembolism (VTE), encompassing both deep vein thrombosis (DVT) and pulmonary embolism (PE), remains a significant cause of morbidity and mortality worldwide with an incidence of 1–2 cases per 1000 [1–3]. While traditional risk factors such as immobility [4], surgery [5], and malignancy [6] are well-established, less than half of cases have no major provoking factors [7]. Factors such as obesity [8] and age [9] increase the risk of unprovoked VTE.

Metabolic syndrome (MetS), characterized by abdominal obesity, insulin resistance, dyslipidemia, and hypertension, is associated with increased risk of cardiovascular disease (CVD) [10]. It has a global prevalence of 13–31%, increasing with the country’s level of income [11]. Among the individual components (MetS factors), all are common risk factors for CVD, but abdominal obesity appears most strongly linked to VTE [12–14]. The mechanisms by which obesity contributes to VTE are thought to involve venous stasis and alterations in the coagulation system, including impaired fibrinolytic activity and elevated plasma levels of clotting factors and inflammatory factors [15].

Several studies have investigated the impact of MetS on the risk of VTE. A prospective cohort study with a mean follow-up time of 12.3 years found an association between MetS and VTE with similar risk in both sexes [16]. In contrast, another similar cohort study found this association only in men [17]. Additionally, both studies concluded that abdominal obesity is essential for any association between MetS and VTE to exist. A meta-analysis from 2014 contrasted these results by including case-control studies showing a persistent association, irrespective of abdominal obesity and consistent across sexes [18]. More recently, a large-scale Korean study of nearly 8 million individuals found that MetS does not increase the risk of VTE but confirmed abdominal obesity as a significant independent risk factor [19]. The few studies that have explored how the association between MetS and VTE varies by sex are inconsistent. Comprehensive and fully sex-stratified analyses of the relationship between MetS and VTE, including its subtypes PE and DVT, are lacking. In summary, while abdominal obesity has been consistently linked to VTE risk, the contribution of MetS as a whole syndrome, and the extent to which this relationship varies by sex, remains inconclusive.

The aim of the present study was to investigate the association between MetS and VTE, in a Swedish cohort of middle-aged and older individuals from southern Sweden. Additional aims were to examine how this association differ between men and women, and to evaluate the importance of the various MetS components with and without abdominal obesity.

## Methods

### Study population

The Malmö Preventive Project (MPP) is a prospective cohort study which started in 1974 in the city of Malmö, in the south of Sweden. The program invited men and women between the ages of 35 and 70 residing in the urban area of Malmö to participate in a comprehensive health screening.

In total, 22,444 men and 10,902 women were enrolled between 1974 and 1982. The study design has been described in previous studies [20]. Data for the present study was collected during a re-examination including physical examination and laboratory tests conducted between 2002 and 2006, inviting participants from the baseline who were still alive and still living in the area of Malmö. Out of approximately 25,000 individuals invited to the re-examination, 18,240 participated [21]. Participants were followed in national registers until 31 December 2018 [22].

Totally 234 participants were excluded due to missing data on MetS factors, and 82 participants due to additional missing information. Additionally, 3533 men and 1603 women were excluded due to conditions before baseline which may affect the risk of VTE [23–28]: prevalent VTE, type 1 diabetes, malignancy, or arterial cardiovascular disorders (Supplementary Table 1). Moreover, impaired fasting glucose (IFG) is included in the definition of METS. Type 1 diabetes may therefore affect the METS definition, which was another reason for excluding patients with Type 1 diabetes.

International Statistical Classification of Diseases and Related Health Problems (ICD) codes from the Swedish Hospital Discharge Register and the Hospital Outpatient Register (both part of the National Patient Register) were used to collect data on VTE, PE, and DVT diagnoses as well as diagnoses for exclusions [22, 29–31] (Supplementary Table 1). Cancer diagnoses were collected from the Swedish Cancer Register [32]. Although several provoking factors were excluded, information on recent surgery or immobilization was not collected.

### Measurements and definitions

During a first visit as part of the re-examination, participants received oral information about the study and underwent blood sampling following an overnight fast [33]. Laboratory analyses included fasting plasma glucose (FPG), serum (s-) total cholesterol, s-triglycerides, and s-high-density lipoprotein (HDL) cholesterol (Beckman Coulter LX20, Beckman Coulter Inc., Brea, USA). Low-density lipoprotein (LDL) cholesterol was calculated using the Friedewald formula [34]. Approximately one week later, at a follow-up visit, trained nurses measured blood pressure twice in the supine position after five minutes of rest. Body measurements were recorded, including height, weight, waist and hip circumference. Participants also completed a questionnaire covering lifestyle factors, self-rated health, brief medical history, and current medications. For individuals with an FPG of ≥ 7.0 mmol/L at the first visit, additional blood samples were taken during the second visit.

MetS was defined according to the National Cholesterol Education Program Adult Treatment Panel III (NCEP ATPIII), although with a slightly lower threshold for fasting glucose [35]. By this definition MetS is present if three or more of the following factors are met: abdominal obesity, elevated triglycerides, low HDL-cholesterol, hypertension, or IFG. Abdominal obesity was defined as a waist circumference of ≥ 102 cm in men and ≥ 88 cm in women. Elevated triglycerides were defined as triglycerides ≥ 150 mg/dL (1.7 mmol/L) or on triglyceride-lowering medication. Low HDL-cholesterol was defined as < 40 mg/dL (1.03 mmol/L) for men and < 50 mg/dL (1.30 mmol/L) for women, or on drug treatment for reduced HDL-cholesterol. Hypertension was defined as systolic blood pressure ≥ 130 mmHg, or diastolic blood pressure ≥ 85 mmHg, or on blood pressure-lowering medication. IFG was defined as ≥ 100 mg/dL (5.6 mmol/L) or on drug treatment for elevated glucose. Certain analyses were stratified by abdominal obesity, in which MetS was defined as three or more of the remaining four MetS factors (elevated triglycerides, low HDL-cholesterol, hypertension, and IFG).

### Outcomes

The primary outcome was the risk of VTE incident in people with MetS versus those without. Secondary outcomes were the risk of PE and DVT in people with MetS versus those without. Additional outcomes were: change in VTE risk with and without abdominal obesity, and difference in risk between sexes.

### Statistical analysis

Statistical analysis was conducted using SPSS version 29.0 (IBM Corp., Armonk, NY, USA). Due to lack of functionality in SPSS, Schoenfeld residuals were tested in Stata version 18.0 (StataCorp LLC, College Station, TX, USA). When calculating differences between groups, chi-square was used for categorical variables and Student’s t-test for continuous variables. Normality was tested using Kolmogorov-Smirnov and Shapiro-Wilk scores, in combination with reviewing histogram plots. Cox proportional hazard regression models with hazard ratios (HR) and 95% confidence intervals (CI) were used to study the influence of MetS factors on the incidence of VTE, PE, and DVT. P-values < 0.05 were considered statistically significant. Participants were followed from re-examination until December 31st, 2018, or censored from further follow-up in the case of emigration, death, diagnosis of cancer, or arterial thromboembolism (ATE) prior to VTE, PE, or DVT. A diagnosis of VTE, PE, or DVT also led to censoring from further follow-up, meaning only initial events were registered. Models were stratified by sex and performed as univariate analyses, in addition to adjusted models including potential confounders: age [36], height [37], and smoking [38]. No significant differences were found when testing a model only adjusted for age. The variable for MetS was dichotomized, as by its definition (0–2 factors or ≥ 3 factors). Additionally, a categorical variable was created with increasing MetS factors (0, 1, 2, or ≥ 3). To clarify the importance of waist circumference, the effects of an increasing number of MetS factors were also studied after stratification by abdominal obesity. Both the groups with and the groups without abdominal obesity were compared to a “healthy” reference group (no abdominal obesity and no other MetS factors). Additionally, a quartile-based analysis of waist circumference was performed separately for men and women to further explore its significance. The proportional hazards assumption was verified by visual inspection of log-minus-log plots and by formal testing with Schoenfeld residuals and was found not to be violated.

## Results

Among 7806 men there were 414 incident VTE events during a total follow-up time of 82408.38 person-years (mean 10.6 years), resulting in an incidence rate of 5.02 per 1000 person-years. In 4982 women there were 316 incident VTE events during a total follow-up time of 58408.98 person-years (mean 11.7 years), corresponding to an incidence rate of 5.41 per 1000 person-years. Characteristics of the study population, split by sex, are presented in Table 1.

**Table 1:** Characteristics of the study population

	Mean n=7806	Women n=4982	p
	n (%)^a^	n (%) ^a^	
Nr of MetS factors			< 0.001
0	520 (6.7)	461 (9.3)	
1	1837 (23.5)	1395 (28.0)	
2	2103 (26.9)	1168 (23.4)	
3	1642 (21.0)	937 (18.8)	
4	1113 (14.3)	640 (12.8)	
5	591 (7.6)	381 (7.6)	
MetS	3346 (42.9)	1958 (39.3)	< 0.001
MetS in persons without obesity^b^	1223 (24.0)	462 (16.8)	< 0.001
MetS in persons with obesity^c^	1247 (46.0)	883 (39.5)	< 0.001
Abdominal obesity	2713 (34.8)	223 (44.9)	< 0.001
Hypertension	6620 (84.8)	4027 (80.8)	< 0.001
Low HDL cholesterol	2497 (32.0)	1739 (34.9)	< 0.001
Hypertriglyceridemia	2408 (30.8)	1341 (26.9)	< 0.001
IFG	4138 (53.0)	1665 (33.4)	< 0.001
Smoking			< 0.001
Current smoker	1176 (15.7)	689 (15.1)	
Previous smoker	4095 (54.8)	1807 (39.7)	
Age, mean ± SD	67.3 (6.0)	69.1 (4.9)	< 0.001
Height, mean ± SD	175.7 (6.7)	162.1 (6.0)	< 0.001
BMI, kg/m^2^, mean ± SD	27.1 (3.7)	26.8 (4.6)	< 0.001

^a^n (%) unless stated otherwise.

^b^≥3 MetS factors excluding abdominal obesity, in persons without abdominal obesity.

^c^≥3 MetS factors excluding abdominal obesity, in persons with abdominal obesity.

HDL: high-density lipoprotein. MetS: metabolic syndrome. MetS factor: risk factor part of the metabolic syndrome. BMI: body mass index. IFG: impaired fasting glucose. Chi-Square test for categorical variables, two-tailed t-test for continuous variables. Level of significance: p <0.05.

The prevalence of MetS was greater in men than in women (p = < 0.001). Hypertension, hypertriglyceridemia, and IFG were more common in men, while women showed higher rates of abdominal obesity and low HDL (p = < 0.001). Ages ranged from 56 to 83 years, and women were significantly older than men (69.1 versus 67.3 years). Incident VTE was more common among women (*p* = 0.014).

Neither univariate analyses nor analyses adjusted for age, height, and smoking showed an association between MetS (dichotomized as 0–2 factors versus ≥ 3 factors) and increased risk of VTE (Table 2).

**Table 2:** Hazard ratios with 95% confidence intervals for VTE in relation to MetS and individual MetS factors

	Univariate				Age, height, and smoking adjusted			
	Men		Women		Men		Women	
	HR	(95% CI)	HR	(95% CI)	HR	(95% CI)	HR	(95% CI)
MetS^a^	1.14	(0.94–1.39)	1.22	(0.98–1.53)	1.10	(0.90–1.34)	1.15	(0.91–1.45)
1 MetS factor^b^	1.10	(0.71–1.71)	1.49	(0.87–2.57)	1.07	(0.68–1.68)	1.44	(0.81–2.57)
2 MetS factors^b^	1.12	(0.73–1.74)	2.38	(1.40–4.05)	1.11	(0.71–1.73)	2.29	(1.30–4.04)
≥ 3 MetS factors^b^	1.26	(0.83–1.91)	2.13	(1.27–3.58)	1.19	(0.77–1.82)	1.96	(1.13–3.41)
Abdominal obesity	1.29	(1.06–1.57)	1.81	(1.45–2.26)	1.21	(0.98–1.48)	1.80	(1.43–2.27)
Hypertension	1.04	(0.79–1.35)	1.35	(0.995–1.84)	0.96	(0.73–1.26)	1.33	(0.96–1.86)
Low HDL cholesterol	0.98	(0.80–1.21)	0.98	(0.77–1.23)	0.98	(0.79–1.21)	0.95	(0.74–1.20)
Hypertriglyceridemia	0.81	(0.65–1.01)	1.12	(0.88–1.43)	0.85	(0.68–1.06)	1.06	(0.82–1.36)
IFG	1.07	(0.88–1.30)	1.31	(1.04–1.64)	1.04	(0.85–1.27)	1.26	(0.999-1.60)

^a^≥3 MetS factors compared to 0–2 MetS factors.

^b^Compared to a reference of zero MetS factors.

VTE: venous thromboembolism, MetS: metabolic syndrome, MetS factor: risk factor part of the metabolic syndrome, HDL: high-density lipoprotein, IFG: impaired fasting glucose.

**Fig. 1:**
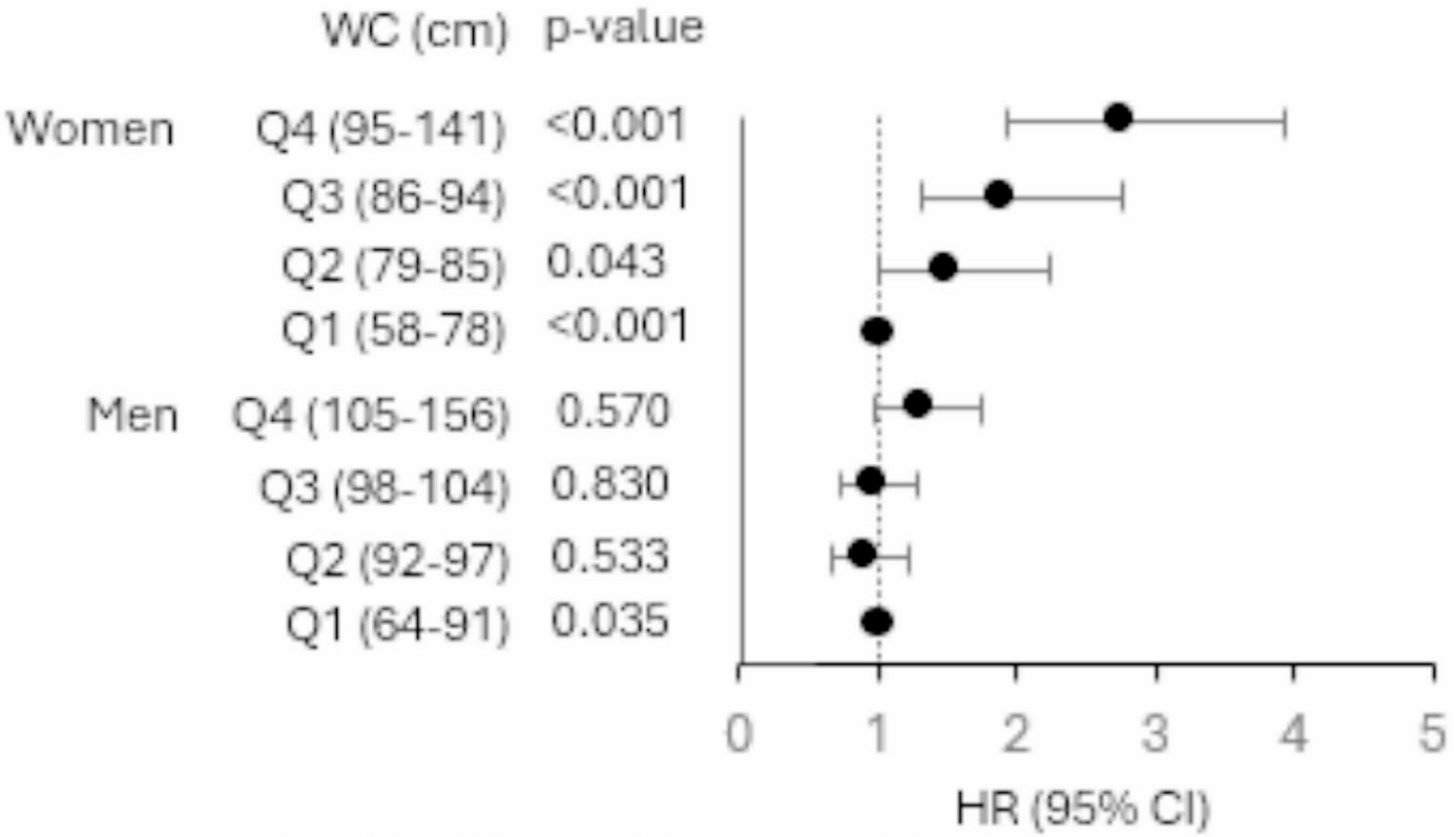
Adjusted hazard ratios with 95% confidence intervals for VTE across quartiles (Q1-Q4) of waist circumference, stratified by sex. VTE: venous thromboembolism. cm: centimeters. Reference group = Q1

However, women with two risk factors (adjusted HR 2.29; 95% CI 1.30–4.04) or a minimum of three risk factors (adjusted HR 1.96; 95% CI 1.13–3.41) showed a greater risk of VTE compared to those with zero risk factors, in both univariate and adjusted models. This pattern was not found in men. Abdominal obesity was linked to increased risk of VTE in women across all models, but only in univariate analysis in men. However, in a separate analysis excluding height from adjustments, the association between abdominal obesity and VTE risk in men reached a statistically significant level (Supplementary Table 2). In a quartile-based model, increased waist circumference showed an increased risk of VTE in a dose-dependent manner among females (Fig. 1). The adjusted HRs of the top quartiles (Q2, Q3, and Q4) compared to the lowest (Q1) were 1.51 (95% CI 1.01–2.25), 1.91 (95% CI 1.32–2.77), and 2.75 (95% CI 1.92–3.94), respectively. In the male quartile-based model, increased waist circumference showed no increased risk of VTE. None of the other individual MetS factors were associated with VTE risk in adjusted models.

In subanalyses, PE and DVT showed similar associations as VTE, with the addition of high f-glucose (HR 1.73; 95% CI 1.18–2.52) and hypertension (HR 1.94; 95% CI 1.04–3.64) reaching a significant association with DVT in women (Supplementary Tables 3 and 4). Also, there were no associations between PE and multiple MetS factors, as seen for VTE and DVT in women. Abdominal obesity was associated with an increased risk of PE (HR 1.77; 95% CI 1.26–2.50) and DVT (HR 2.02; 95% CI 1.37–2.97) in women.

**Fig. 2:**
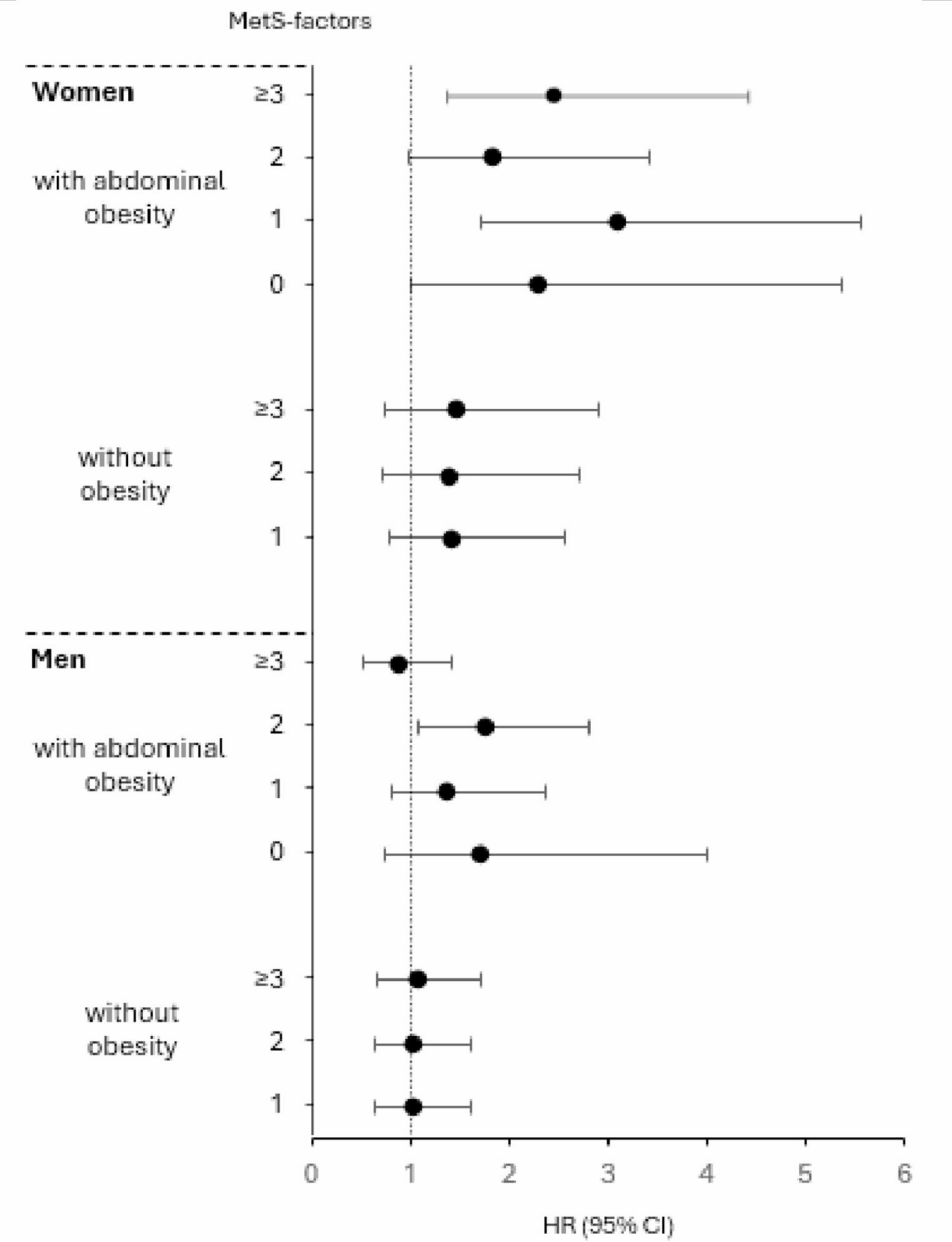
Adjusted hazard ratios with 95% confidence intervals for VTE compared to reference group (HR =1) of healthy individuals (persons without abdominal obesity and without other MetS factors). VTE: venous thromboembolism. MetS: metabolic syndrome. MetS factor: risk factor part of the metabolic syndrome

Figure 2 Adjusted hazard ratios with 95% confidence intervals for VTE compared to reference group (HR = 1) of healthy individuals (persons without abdominal obesity and without other MetS factors).

When stratifying by abdominal obesity and analyzing participants without abdominal obesity using a four-factor MetS definition, no association with VTE risk was found (Supplementary Table 5). As visualized in Fig. 2, the HR for VTE was generally higher in groups with abdominal obesity, compared to those without. In men with abdominal obesity, only two MetS factors were significantly associated (Fig. 2 and Supplementary Table 6).

Women with abdominal obesity and one or three MetS factors showed a significant association with VTE. We found no association between smoking and any type of thromboembolism, except among men without abdominal obesity and the risk of PE (HR 2.06; 95% CI 1.18–3.60).

To test the robustness of the findings, a sensitivity analysis was conducted including participants otherwise excluded due to prevalent disease. In both men and women, MetS became significantly associated with VTE in both univariate and adjusted models (Supplementary Table 7). However, the delta in HR compared to regular analyses is considered minor, along with a lower confidence interval limit close to 1. Also, analyses on several MetS factors grouped remained similar.

## Discussion

The present cohort study of 12,788 Swedish men and women with a mean follow-up time of 11 years found no association between dichotomously defined MetS and VTE risk, in either sex, both in univariate analyses and after adjusting for potential confounders (age, height, and smoking). Abdominal obesity was associated with VTE risk only in women, as shown in the adjusted analysis (HR 1.80; 95% CI 1.43–2.27). An overall increased risk was observed among individuals with abdominal obesity in stratified analyses, with a more pronounced effect in women. Also, a dose-dependent increased risk of VTE from increased waist circumference was most obvious in women. This suggests that the higher VTE risk seen in women with multiple MetS factors compared to zero is largely attributable to abdominal obesity. Supporting this interpretation, the other MetS factors showed no significant impact on VTE risk in individuals without abdominal obesity. Moreover, there was no discernible trend of an increasing VTE risk with an increasing number of MetS factors, as was found by Borch et al. [16]

Our results differ somewhat from previous research by Steffen et al. [17], who found MetS to be associated with risk of total VTE (provoked and unprovoked) among men only, and Borch et al. [16], who found MetS to be associated with provoked VTE but not unprovoked, in both sexes. Different inclusion/exclusion criteria might explain the divergent findings, which are also seen in our sensitivity analysis, showing slightly altered HRs after including participants with baseline comorbidities. Other demographic profiles and genetic backgrounds may also cause some of the divergent results. Additionally, adjustments in Cox regression models differ between studies. As an example, Park et al. [19] found an association between MetS and VTE risk in a model adjusted for sex, age, smoking, exercise status, family history of hypertension, stroke, heart disease, and diabetes, but after including BMI and cholesterol, among other variables, associations were attenuated. This exemplifies the challenge in comparing these different study results.

Although our adjusted analysis only found women with abdominal obesity to have an increased risk of VTE, men also showed an increased risk in univariate analysis, but associations were attenuated after adjusting for potential confounders. Height has been identified as a risk factor for VTE in both men and women, although results are more consistent for men [39–42]. Thus, adjusting for height in men may attenuate the association between waist circumference and VTE, as some of the risk attributed to abdominal obesity may actually reflect risk driven by greater body size or stature. Such attenuation was confirmed in a separate analysis of our data (Supplementary Table 2). This supports the idea that abdominal obesity plays a more significant role in VTE risk among women. Other studies have found that abdominal obesity carries a risk in both sexes [12, 14, 43, 44], including one that also adjusted for height in their models [45]. Similar to our results, Brink et al. [46] found abdominal obesity linked to PE and DVT in women but not in men, in a Swedish cohort using similar exclusion criteria and also adjusting for height. When they included patients with cancer before baseline and cancer-related VTE incidents, the risk was also present in men. Hence, they hypothesize that the association between men with abdominal obesity and the risk of DVT and PE may be mediated through cancer-related pathways. Obesity is a risk factor for cancer, and it promotes carcinogenesis through interlocking of not only metabolic but also inflammatory, immune, and hormonal pathways. Moreover, biological plausibility and convergent epidemiology support obesity as a modifiable cancer risk factor [47]. Given the mixed findings in previous research, it is difficult to conclude whether abdominal obesity independently increases risk in men, or if the observed association is confounded by other factors such as cancer or height. Our results showed that the other individual MetS factors (excluding waist) have little impact on VTE risk, in alignment with previous research stating that traditional cardiovascular risk factors lack an association with risk of VTE [13, 28]. However, we found that IFG and hypertension increase the risk of DVT specifically in women. Borch et al. [16] observed similar associations, but only in univariate analysis and with both sexes combined.

The pathophysiological link between obesity and VTE is multifactorial. Increased intra-abdominal pressure reduces venous return from the lower limbs and slows venous flow, promoting venous stasis [15]. Obesity is also associated with elevated plasminogen activator inhibitor-1, which inhibits fibrinolysis and promotes a prothrombotic state [15, 48]. Additional haemostatic changes include higher circulating levels of fibrinogen and factor VIII [15]. Additionally, the inflammatory activity of adipose tissue, causing increased levels of C-reactive protein, IL-6, and TNF-α, contributes to oxidative stress, which in turn promotes platelet aggregation, endothelial dysfunction, and thrombus formation [15]. These haemostatic, inflammatory, endothelial, and flow-related mechanisms may explain why adiposity appears to be the dominant metabolic factor associated with VTE risk. Some previous research reports stronger associations between circulating levels of fibrinogen and C-reactive protein in women with obesity than in men [49], which may partly explain our finding that abdominal obesity was linked to thrombotic risk predominantly in women. Our results add to the growing body of evidence on obesity’s adverse health effects and underscore the potential clinical benefits of early lifestyle interventions. Clinicians should also remain mindful of potential sex-specific differences that may not be captured by standard risk assessments.

The population-based cohort used in this study offers several strengths, including a large sample size, long-term follow-up, and outcome data derived from national registries. A case validity of 95% has been reported in a study examining VTE in the National Patient Register [31]. The overall positive predictive values of diagnoses in the Swedish Hospital Discharge Register are estimated to be about 85–95% [22]. Sex stratification and separate analyses of PE and DVT outcomes provide a more detailed assessment of the associations.

A limitation of our study is that MetS factors were only collected at baseline (re-examination) and not updated over time. This limits our ability to account for changes in metabolic health during follow-up. However, weight gain over time is more common than weight loss [50]. Another limitation is the lack of information on physical activity and cardiorespiratory fitness. We had no access to detailed information about medication for blood lipids, blood pressure treatment, and drug treatment for elevated glucose, which is a limitation. We had no information about anticoagulant treatment, which is a limitation. However, we excluded patients with VTE or arterial cardiovascular disorders at baseline, i.e. individuals at increased risk of receiving anticoagulation treatment. Different study designs, MetS definitions, and exclusion criteria might make risk estimates difficult to compare across studies. As an example, a meta-analysis by Abuduhalike et al. [51] used BMI instead of waist circumference as obesity measurement, hindering a comparison of results. Although major factors like cancer and CVD were excluded, our study lacked data on recent surgery and immobilization. Thus, our definition of VTE is not strictly “unprovoked” or “idiopathic” as defined by some previous authors [16–18], making the findings less generalizable. Finally, the cohort included more men than women, limiting generalizability to a balanced general population.

## Conclusion

MetS is not associated with VTE risk in either sex. Abdominal obesity is associated with increased risk, but mainly in women. The other components of MetS appear to have limited impact on VTE risk.

## Supplementary Information

Below is the link to the electronic supplementary material.

Supplementary Material 1

## Data Availability

The data that support the findings of this study are available upon reasonable request.
