# Supplementary material for: Metabolic syndrome and venous thromboembolism: the role of abdominal obesity and sex differences
Source: J Thromb Thrombolysis. 2026 Mar 6;59(3):541–50. doi: 10.1007/s11239-026-03255-x (PMC13246895; doi:10.1007/s11239-026-03255-x)
Supplement: Supplementary file 1 — Supplementary Material 1 [file 11239_2026_3255_MOESM1_ESM.docx]

**SUPPLEMENTARY MATERIAL**

**Metabolic syndrome and venous thromboembolism: The role of abdominal obesity and sex differences**

Journal of Thrombosis and Thrombolysis

Niklas Brodin^a, b *^, Peter Nymberg^a, b^, Beata Borgström Bolmsjö^a, b^, Peter J Svensson ^c^, Johan Elf ^c^, Bengt Zöller^a, b**^, Susanna Calling^a, b**^

^a^ University Clinic Primary Care, Skåne University Hospital, Region Skåne, Sweden.

^b^ Center for Primary Health Care Research, Department of Clinical Sciences, Lund University, Malmö, Sweden.

^c^ Department of hematology, oncology and radiotheraphy, Skåne University Hospital, and departement of translational research, Lund University, both Malmö, Sweden.

*Correspondence to Niklas Brodin, Center for Primary Health Care Research, Department of Clinical Sciences in Malmö, Lund University, Jan Waldenströms gata 35, Skåne University Hospital, 205 02 Malmö, SWEDEN. Tel: +46 40 39 14 00; fax: +46 40 39 13 70; e-mail: [niklas.brodin@med.lu.se](mailto:niklas.brodin@med.lu.se); ORCID iD: 0009-0006-0592-0027

**Shared last authorship

**Supplementary Table 1. ICD codes for incident diagnoses and exclusions**

|  | Descriptions, where relevant | ICD 10 | ICD 9 | ICD 8 | ICD 7 |
| --- | --- | --- | --- | --- | --- |
| type 1 diabetes |  | E103, E104, E104X, E105, E106, E108, E109 |  |  |  |
| VTE (Venous thromboembolism) | Pulmonary embolism with or without infarction, Chronic pulmonary embolism, Pulmonary embolism in pregnancy and puerperium, other venous embolism and thrombosis (Includes embolism and thrombosis of vena cava, renal vein, other specified veins, and unspecified vein), Venous complications in pregnancy and puerperium, Phlebitis and thrombophlebitis, Phlebitis and thrombophlebitis (deep venous thrombosis) of lower limb veins including during pregnancy, childbirth, and the puerperium, Portal vein thrombosis, Cerebral venous thrombosis, Migratory Thrombophlebitis, other venous thrombosis | I26, I636, I676, I80, I81, I82, O222, O223, O225, O229, O082, O870, O871, O873, O879, O882 | 325, 415B, 416W, 437G, 451, 452, 453, 639G, 671C, 671D, 671E, 671F, 671X, 673C | 321, 450, 451, 452, 453, 671, 6739 | 33440, 33450, 463, 464, 465, 466, 58300, 682, 684 |
| DVT (Deep vein thrombosis) | Phlebitis and thrombophlebitis (deep venous thrombosis) of lower limb veins including during pregnancy, childbirth, and the puerperium,  *Excluding superficial vein thrombosis. | I80, O223, O871  *excluding I800 | 451, 671D, 671E  *excluding 451A | 451, 67101, 67102, 67108, 67109 | 463, 682 |
| PE (Pulmonary embolism) | Pulmonary embolism with or without infarction, including in pregnancy and puerperium, Chronic pulmonary embolism, Embolism after abortion | I26, O082, O882 | 415B, 416W, 639G, 673C | 450, 6739 | 465, 684 |
| portal vein thrombosis |  | I81 | 452 | 452 | 58300 |
| superficial thrombophlebitis |  | I800, O222, O870 | 451A, 671C | 67100 |  |
| arterial thrombosis and embolism |  | I74 | 444 | 444 | 454 |
| all peripheral artery disease |  | I702, I73 | 440C, 443X | 44020, 443, 445 | 453, 455 |
| aortic aneurysm |  | I71 | 441 | 441 | 451 |
| abdominal aortic aneurysm |  | I713, I714, I715, I716 | 441D, 441E | 44120 |  |
| thoracic aortic aneurysm |  | I711, I712, I715, I716 | 441B, 441C | 44110 |  |
| aortic dissection |  | I710 | 441A | 44100 | 45120 |
| aortic stenosis |  | I350, I352 | 424B, 4241 | 42410,42411, 42419 |  |
| atrial fibrillation or flutter event |  | I48 | 427D | 42792 | 43312, 43313 |
| malignancy |  | C00-C97 | 140-208 | 140-209 | 140-205 |
| coronary event |  | I24, I252, I20, I251, I253-I259 | 410-414 | 410,411,41201,41291,41292,413,414 |  |
| stroke |  | I60, I61, I63, I64 | 430,431,434,436 | 430,43100,43109,43190,433,434,43600,43699 |  |
| heart failure |  | I50, I110 | 428 | 42700,42710,42899 |  |
| individuals on warfarin drug treatment | ATC (Anatomical Therapeutic Chemical Classification System) code B01AA03 | - | - | - | - |

**Supplementary Table 2. Hazard ratios with 95% confidence intervals for VTE in relation to abdominal obesity.**

|  | Age and smoking adjusted | | | |  | Age, height, and smoking adjusted | | | |
| --- | --- | --- | --- | --- | --- | --- | --- | --- | --- |
|  | Men | |  | |  | Men | |  | |
|  | HR | (95% CI) |  |  |  | HR | (95% CI) |  |  |
| Abdominal obesity | 1.26 | (1.03-1.54) |  |  |  | 1.21 | (0.98-1.48) |  |  |
|  |  |  |  |  |  |  |  |  |  |
| VTE: venous thromboembolism. | | | | | | | | | |

Clarification to supplementary table: Abdominal obesity’s influence on VTE risk, tested in two different adjustment models.

**Supplementary Table 3. Hazard ratios with 95% confidence intervals for PE in relation to MetS and individual MetS factors.**

|  | Univariate | | | |  | Age, height, and smoking adjusted | | | |
| --- | --- | --- | --- | --- | --- | --- | --- | --- | --- |
|  | Men | | Women | |  | Men | | Women | |
|  | HR | (95% CI) | HR | (95% CI) |  | HR | (95% CI) | HR | (95% CI) |
| MetS^a^ | 1.14 | (0.85-1.54) | 1.18 | (0.85-1.64) |  | 1.11 | (0.81-1.50) | 1.08 | (0.77-1.52) |
| 1 MetS factor^b^ | 1.22 | (0.63-2.35) | 1.29 | (0.59-2.81) |  | 1.24 | (0.62-2.48) | 1.03 | (0.47-2.26) |
| 2 MetS factors^b^ | 0.92 | (0.47-1.81) | 2.37 | (1.11-5.02) |  | 0.98 | (0.49-1.97) | 1.98 | (0.93-4.21) |
| >3 MetS factors^b^ | 1.20 | (0.64-2.26) | 1.94 | (0.93-4.06) |  | 1.21 | (0.62-2.34) | 1.50 | (0.71-3.14) |
| Abdominal obesity | 1.28 | (0.95-1.74) | 1.87 | (1.34-2.60) |  | 1.18 | (0.86-1.63) | 1.77 | (1.26-2.50) |
| Hypertension | 1.03 | (0.68-1.56) | 1.24 | (0.80-1.91) |  | 0.98 | (0.64-1.49) | 1.08 | (0.68-1.71) |
| Low HDL cholesterol | 0.92 | (0.67-1.28) | 0.92 | (0.65-1.30) |  | 0.93 | (0.66-1.29) | 0.90 | (0.63-1.29) |
| Hypertriglyceridemia | 0.75 | (0.53-1.06) | 1.05 | (0.73-1.50) |  | 0.82 | (0.58-1.16) | 1.02 | (0.70-1.48) |
| IFG | 0.99 | (0.74-1.34) | 1.12 | (0.79-1.57) |  | 1.00 | (0.73-1.35) | 1.08 | (0.76-1.54) |
|  |  |  |  |  |  |  |  |  |  |
| ^a^ ≥3 MetS factors compared to 0-2 MetS factors. ^b^ Compared to a reference of zero MetS factors. PE: pulmonary embolism. MetS: metabolic syndrome. MetS factor: risk factor part of the metabolic syndrome. HDL: high-density lipoprotein. IFG: impaired fasting glucose. | | | | | | | | | |

Clarification to supplementary table: Shows similar analysis as for VTE but for PE instead.

**Supplementary Table 4. Hazard ratios with 95% confidence intervals for DVT in relation to MetS and individual MetS factors**

|  | Univariate | | | |  | Age, height, and smoking adjusted | | | |
| --- | --- | --- | --- | --- | --- | --- | --- | --- | --- |
|  | Men | | Women | |  | Men | | Women | |
|  | HR | (95% CI) | HR | (95% CI) |  | HR | (95% CI) | HR | (95% CI) |
| MetS^a^ | 1.26 | (0.93–1.71) | 1.24 | (0.86–1.78) |  | 1.22 | (0.89–1.68) | 1.22 | (0.84-1.78) |
| 1 MetS factor^b^ | 1.26 | (0.58-2.71) | 1.73 | (0.67–4.51) |  | 1.17 | (0.54–2.53) | 2.41 | (0.72–8.01) |
| 2 MetS factors^b^ | 1.43 | (0.67-3.03) | 3.03 | (1.19–7.73) |  | 1.36 | (0.64–2.91) | 4.19 | (1.28–13.68) |
| >3 MetS factors^b^ | 1.64 | (0.79-3.40) | 2.61 | (1.04–6.53) |  | 1.51 | (0.73–3.15) | 3.52 | (1.09–11.30) |
| Abdominal obesity | 1.30 | (0.95–1.77) | 1.80 | (1.25–2.58) |  | 1.24 | (0.90–1.72) | 2.02 | (1.37-2.97) |
| Hypertension | 1.23 | (0.78–1.93) | 1.82 | (1.04–3.18) |  | 1.13 | (0.72–1.78) | 1.94 | (1.04–3.64) |
| Low HDL cholesterol | 1.14 | (0.83–1.57) | 0.99 | (0.68–1.45) |  | 1.13 | (0.81–1.57) | 0.97 | (0.66-1.45) |
| Hypertriglyceridemia | 0.89 | (0.63–1.25) | 1.06 | (0.71–1.59) |  | 0.92 | (0.65–1.30) | 1.03 | (0.68-1.56) |
| IFG | 1.22 | (0.90–1.67) | 1.79 | (1.25–2.57) |  | 1.18 | (0.86–1.63) | 1.73 | (1.18–2.52) |
|  |  |  |  |  |  |  |  |  |  |
| ^a^ ≥3 MetS factors compared to 0-2 MetS factors. ^b^ Compared to a reference of zero MetS factors. DVT: deep vein thrombosis. MetS: metabolic syndrome. MetS factor: risk factor part of the metabolic syndrome. HDL: high-density lipoprotein. IFG: impaired fasting glucose. | | | | | | | | | |

Clarification to supplementary table: Shows similar analysis as for VTE but for DVT instead.

**Supplementary Table 5. Hazard ratios with 95% confidence intervals for VTE in relation to MetS in persons without abdominal obesity**

|  | Univariate | | | |  | Age, height, and smoking adjusted | | | |
| --- | --- | --- | --- | --- | --- | --- | --- | --- | --- |
|  | Men | | Women | |  | Men | | Women | |
|  | HR | (95% CI) | HR | (95% CI) |  | HR | (95% CI) | HR | (95% CI) |
| MetS^a^ | 1.07 | (0.81–1.43) | 1.18 | (0.77–1.83) |  | 1.09 | (0.81–1.46) | 1.13 | (0.72-1.78) |
|  |  |  |  |  |  |  |  |  |  |
| ^a^ ≥3 MetS factors compared to 0-2 MetS factors. VTE: venous thromboembolism. MetS: metabolic syndrome. | | | | | | | | | |

**Supplementary Table 6. Hazard ratios with 95% confidence intervals for VTE in relation to MetS factors after stratification by abdominal obesity**

|  |  | MetS factors^a^ | HR^b^ | 95% CI | |
| --- | --- | --- | --- | --- | --- |
|  |  |  |  |  |  |
| Men | without obesity | 1 | 1.02 | 0.65 | 1.62 |
|  |  | 2 | 1.02 | 0.64 | 1.62 |
|  |  | ≥3 | 1.07 | 0.66 | 1.72 |
|  |  |  |  |  |  |
|  | with abdominal obesity | 0 | 1.72 | 0.74 | 4.01 |
|  |  | 1 | 1.38 | 0.81 | 2.37 |
|  |  | 2 | 1.75 | 1.09 | 2.81 |
|  |  | ≥3 | 0.87 | 0.53 | 1.42 |
|  |  |  |  |  |  |
| Women | without obesity | 1 | 1.43 | 0.80 | 2.57 |
|  |  | 2 | 1.39 | 0.72 | 2.70 |
|  |  | ≥3 | 1.47 | 0.74 | 2.90 |
|  |  |  |  |  |  |
|  | with abdominal obesity | 0 | 2.31 | 0.995 | 5.37 |
|  |  | 1 | 3.10 | 1.72 | 5.56 |
|  |  | 2 | 1.83 | 0.98 | 3.41 |
|  |  | ≥3 | 2.47 | 1.38 | 4.42 |
|  |  |  |  |  |  |
| ^a^ Compared to healthy individuals (persons without abdominal obesity and without any MetS factors). ^b^ Age, height, and smoking adjusted. MetS: metabolic syndrome. MetS factor: risk factor part of the metabolic syndrome. VTE: venous thromboembolism. | | | | | |

Clarification to supplementary table: Shows exact numbers that are visualized in figure 2 forest plot.

**Supplementary Table 7. Sensitivity analysis. Hazard ratios with 95% confidence intervals for VTE in relation to MetS factors**

|  | Univariate | | | |  | Age, height, and smoking adjusted | | | |
| --- | --- | --- | --- | --- | --- | --- | --- | --- | --- |
|  | Men (n=11339) | | Women (n=6585) | |  | Men (n=11339) | | Women (n=6585) | |
|  | HR | (95% CI) | HR | (95% CI) |  | HR | (95% CI) | HR | (95% CI) |
| MetS^a^ | 1.21 | **1.05–1.41** | 1.32 | **1.11-1.58** |  | 1.17 | **1.01-1.36** | 1.26 | **1.05-1.52** |
| 1 MetS factor^b^ | 0.97 | 0.68–1.40 | 1.33 | 0.85-2.08 |  | 0.97 | 0.68–1.44 | 1.24 | 0.78-1.98 |
| 2 MetS factors^b^ | 1.07 | 0.75–1.52 | 2.11 | 1.36-3.26 |  | 1.08 | 0.75–1.55 | 1.87 | 1.18-2.95 |
| >3 MetS factors^b^ | 1.24 | 0.89–1.73 | 2.09 | 1.37-3.18 |  | 1.21 | 0.86–1.72 | 1.83 | 1.18-2.84 |
|  |  |  |  |  |  |  |  |  |  |
| ^a^ ≥3 MetS factors compared to 0-2 MetS factors. ^b^ Compared to a reference of zero MetS factors. VTE: venous thromboembolism. MetS: metabolic syndrome. MetS factor: risk factor part of the metabolic syndrome. Values presented in bold were statistically significant in sensitivity analyses but not in main analyses. | | | | | | | | | |

Clarification to supplementary table: Analyses that include previously excluded participants (due to prevalent disease).
